# Autophagic Organelles in DNA Damage Response

**DOI:** 10.3389/fcell.2021.668735

**Published:** 2021-04-12

**Authors:** Jeongha Kim, Sungmin Lee, Hyunwoo Kim, Haksoo Lee, Ki Moon Seong, HyeSook Youn, BuHyun Youn

**Affiliations:** ^1^Department of Integrated Biological Science, Pusan National University, Busan, South Korea; ^2^Laboratory of Low Dose Risk Assessment, National Radiation Emergency Medical Center, Korea Institute of Radiological and Medical Sciences, Seoul, South Korea; ^3^Department of Integrative Bioscience and Biotechnology, Sejong University, Seoul, South Korea; ^4^Department of Biological Sciences, Pusan National University, Busan, South Korea

**Keywords:** DNA damage response, mitophagy, ER-phagy, ribophagy, therapeutic approach

## Abstract

Autophagy is an important subcellular event engaged in the maintenance of cellular homeostasis *via* the degradation of cargo proteins and malfunctioning organelles. In response to cellular stresses, like nutrient deprivation, infection, and DNA damaging agents, autophagy is activated to reduce the damage and restore cellular homeostasis. One of the responses to cellular stresses is the DNA damage response (DDR), the intracellular pathway that senses and repairs damaged DNA. Proper regulation of these pathways is crucial for preventing diseases. The involvement of autophagy in the repair and elimination of DNA aberrations is essential for cell survival and recovery to normal conditions, highlighting the importance of autophagy in the resolution of cell fate. In this review, we summarized the latest information about autophagic recycling of mitochondria, endoplasmic reticulum (ER), and ribosomes (called mitophagy, ER-phagy, and ribophagy, respectively) in response to DNA damage. In addition, we have described the key events necessary for a comprehensive understanding of autophagy signaling networks. Finally, we have highlighted the importance of the autophagy activated by DDR and appropriate regulation of autophagic organelles, suggesting insights for future studies. Especially, DDR from DNA damaging agents including ionizing radiation (IR) or anti-cancer drugs, induces damage to subcellular organelles and autophagy is the key mechanism for removing impaired organelles.

## Introduction

Human cells receive several DNA lesions on ∼10^13^ cells per day in response to various genotoxic insults (Lindahl and Barnes, 2000). DNA damage, due to its potential for mutagenicity, has been shown to be involved in aging, disease, and cancer development. However, there are also positive aspects of DNA damage: both chemotherapy and radiation therapy are typically known to trigger apoptosis in cancer cells by inducing DNA damage. When DNA damage occurs, cells detect it *via* various mechanisms and monitoring systems, resulting in systematic responses to repair the damage, called DNA damage responses (DDRs). DNA damage is divided into double-strand break (DSB) and single-strand break (SSB). When DSB and SSB occur, DDR occurs to maintain the intracellular environment in a normal manner, and signal transduction begins through protein kinase in both DSB and SSB. Ataxia-telangiectasia mutated (ATM) is involved in DSB, and ATM/Rad3-related (ATR) is involved in SSB. The ATM pathway begins when the MRE11/RAD50/NBS1 (MRN) complex binds to the DSB site and recruits ATM. Recruited ATM phosphorylates substrates such as checkpoint kinase 2 (CHK2) or mediator of DNA damage checkpoint protein 1 (MDC1). In the case of MDC1, it is known that a really interesting new gene (RING) finger protein 8 (RNF8), an E3 ubiquitin ligase, recognizes MDC1 and causes ATM to phosphorylate (Yan et al., 2014; Mirza-Aghazadeh-Attari et al., 2018; Tšuiko et al., 2019). Several studies described that DDR occurs through various mechanisms in subcellular organelles within a cell (Boesch et al., 2011). It has been reported that DDR can progress in four directions: activation of cell cycle checkpoint and transcriptional program, DNA repair, and apoptosis (Sancar et al., 2004). For example, radiotherapy and chemotherapy trigger DNA damage to promote cellular apoptosis or senescence as the consequence of the DDR. Recently, DNA damage emerged as a causal factor of aging. DNA damage has a role in aging and age-related diseases and it is further demonstrated by congenital progeroid syndromes.

The autophagy we usually know is macro-autophagy which is not selective. However, an increasing number of recent studies have drawn that autophagy can be greatly selective (Nakatogawa et al., 2009). In contrast to macro-autophagy, in which cytosolic components are engulfed randomly, selective autophagy targets specific cellular components and packages them into membrane vesicles (Mizushima et al., 2011; Rogov et al., 2014). In selective autophagy, these particular substrates are targeted to the autophagosome *via* specific receptors, and the targeted cargo can include protein aggregates, damaged mitochondria, or pathogens, such as bacteria. Besides, numerous studies have reported the selective autophagic degradation of several organelles, including mitochondria, peroxisomes, lysosomes, endoplasmic reticulum (ER), and the nucleus (Gatica et al., 2018). Since damaged such organelles need to be removed, autophagy machinery is activated. Many factors cause damage to organelles, and in the case of damage by DNA damaging agents, studies are continuing to show that autophagy is activated to control cell fate to maintain homeostasis in cells (Stagni et al., 2020). ATM, the major sensor of DSB, is involved in generating signal transduction by recognizing the damaged area when DNA damage occurs, and autophagy is activated by this ATM pathway (Eliopoulos et al., 2016). Ionizing radiation, one of DNA damaging agents, can induce DSB and is known to act as a trigger of autophagy through AKT signaling (Seiwert et al., 2017). In addition to the suggested DSB-related proteins, it is known that proteins required for SSB, such as Poly ADP-ribose polymerase 1 (PARP1), repair also regulate autophagy (Muñoz-Gámez et al., 2009). Autophagosomes engulf cytoplasmic material and organelles, including mitochondria, peroxisomes, lipid droplets, ribosomes, and parts of the nucleus, as part of the processes also known as mitophagy, pexophagy, lipophagy, ribophagy, and nucleophagy, respectively (Eliopoulos et al., 2016). During the autophagy processes, proteins with the LC3 interacting regions (LIR) in mammals are essential in dragging the organelles to autophagosomes (Anding and Baehrecke, 2017). The specificity of selective autophagy is divided according to the type of receptor and interaction with ubiquitin-like proteins through ubiquitin-like modifiers (UBLs) (Shaid et al., 2013). UBLs are directly involved in the autophagosome nucleation machinery, and this regulatory mechanism is a general selective autophagy mechanism; however, various types of selective autophagy activate specific pathways. For example, the PTEN-induced kinase 1 (PINK1)/Parkin pathway is specific to mitophagy, and pexophagy is associated with peroxisomal targeting signal 1 receptor (PTS1R), while the protein phosphatase 1D (PPM1D) pathway is to related lipophagy (Chu, 2019).

The role of autophagy in managing cellular DNA aberrations has been demonstrated by numerous studies. Currently, autophagic degradation of subcellular organelles, including mitochondria, ER, and ribosome, is involved in the determination of cell fates after DNA damage. In this review, we summarize the molecular mechanisms of subcellular autophagic degradation of organelles induced by DDRs and their contribution to managing DNA damage. In addition, we present the pathological mechanisms of the autophagy-related DDR and its potential as a therapeutic target.

## DDR and Autophagic Organelles

### Mitophagy

Mitochondria are organelles with a double membrane that play a central role in the energy metabolism of cells *via* oxidative phosphorylation. Mitochondria also contain genetic information called mitochondrial DNA (mtDNA) (Henze and Martin, 2003). There are several differences between mtDNA and the nuclear genome. Unlike the linear nuclear genome, the circular mitochondrial genome is not enveloped and it is not packaged into chromatin. Thirteen mitochondrial genes play a pivotal role in the survival of cells that produce the subunits of enzymes necessary for oxidative phosphorylation; however, the mitochondrial genome is very small and cannot produce all functionally necessary proteins. As a result, mitochondria are highly dependent on imported nuclear gene products (Taanman, 1999). The mitochondrial genome is exposed to the harmful agents that adversely affect the nuclear genome. Therefore, the DDR in mitochondria can also play a critical role in determining cell fate (Babbar et al., 2020). For this reason, the regulation of mitochondrial homeostasis through mitochondrial biogenesis, bioenergetics, dynamics, mitophagy, and DNA status is important from a cellular perspective (Yu et al., 2017).

Mitophagy is known as the process of removing mitochondria through the autophagy that regulates the homeostasis of mitochondria by eliminating dysfunctional mitochondria. Cell damage that changes energy requirements and developmental changes can all induce mitophagy (Hirota et al., 2015). When gemcitabine, a drug that destroys mtDNA, is used, mitochondrial dysfunction occurs and mitophagy pathway is activated (Inamura et al., 2019). As new mitophagy-related pathways have been demonstrated, the importance of mitophagy is emerging. Mitophagy is regulated by the p53-Spata18 axis (Dan et al., 2020), the PINK1/Parkin pathway, and BCL2 Interacting Protein 3 Like (BNIP3L)/NIX and FUN14 Domain Containing 1 (FUNDC1) related pathway. Among them, the PINK1/Parkin is a key protein for mitophagy signals. Based on the paper, reactive oxygen species (ROS), one of the DNA damaging agents, occurred in the Dox-treated group, activating the PINK1/Parkin pathway and lowering the expression of mitochondrial proteins (Yin et al., 2018). Mitophagy is initiated when an autophagy receptor called PINK1 recognizes damaged mitochondria. In healthy mitochondria, PINK1 is imported into the inner mitochondrial membrane (IMM) through translocase of the outer membrane channel (TOM) and translocase of the inner membrane channel (TIM) by the membrane potential of mitochondria. This leads to the mitochondrial targeting signal being cleaved first by the matrix processing peptidase and then by the protease presenilin-associated rhomboid-like, resulting in the degradation of PINK1 by the proteasome. In damaged mitochondria, the membrane potential of the mitochondria is not maintained. Therefore, PINK1 is not imported into the IMM and accumulates in the membrane of the mitochondria (Nguyen et al., 2016). Accumulated PINK1 recruits an E3 ubiquitin ligase called Parkin that plays a critical role in ubiquitination. When PINK1 and Parkin bind, Parkin ubiquitinates OMM proteins such as mitochondrial profusion protein mitofusin 1 (MFN1), MFN2, voltage dependent anion channels (VDAC), and ras homolog family member T1 (RHOT1/MIRO1) (Shanbhag et al., 2012; Yao et al., 2020), and recruits mitophagy receptor optineurin (OPTN) and nuclear dot protein 52 kDa (NDP52) which is also known as calcium binding and coiled-coil domain 2 (Calcoco2) (Liu et al., 2019). These ubiquitinated proteins act as signals and recruit autophagic machinery to remove damaged mitochondria (McWilliams and Muqit, 2017). Fanconi anemia (FA) pathway genes, known as tumor suppressors, are involved in the repair of damaged nuclear DNA. Also, the association between Parkin and FA pathway genes was revealed, so these proteins may be an important factor in the induction of mitophagy (Sumpter et al., 2016). As such, Parkin and PINK1 are important proteins for mitophagy initiation, and recent paper results that prevent inflammation by removing damaged mitochondria by Parkin-mediated mitophagy suggest that mitophagy is crucial not only in DDR but also in the pathogenesis of other diseases (Sliter et al., 2018). Another mitophagy signal, the BNIP3L/NIX and FUNDC1 pathway, is initiated by FUNDC1 interacting with LC3. FUNDC1 is an outer mitochondrial membrane (OMM) protein, and its interaction with LC3 is regulated by multi-site phosphorylation. This pathway is activated in hypoxic conditions that cause DNA damage and is known to be important as a regulator of the mitochondrial network in the process of cell differentiation (Lampert et al., 2019; Fu et al., 2020).

**FIGURE 1:**
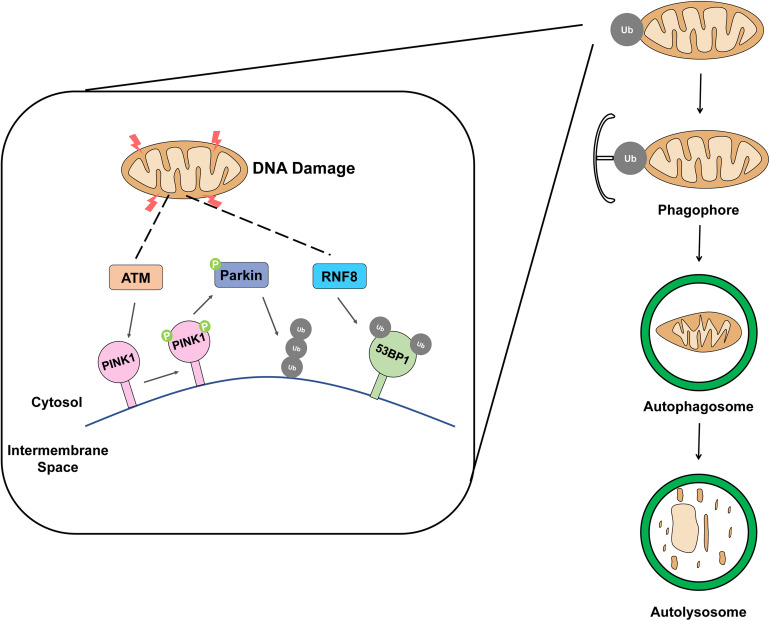
Schematic representation of removing damaged mitochondria by mitophagy. The accumulation of damaged mitochondria is removed by mitophagy when mitochondria are exposed to DNA damage factors such as ionizing radiation, ROS, and drug.

Ataxia-telangiectasia mutated, a serine-threonine protein kinase, is a well-known mediator of DDR which plays a crucial role in maintaining cellular processes, such as hypoxia, oxidative stress, DNA repair, apoptosis, and senescence (Derheimer and Kastan, 2010; Guleria and Chandna, 2016). The intracellular ATM is primarily localized in the nucleus, while smaller amounts can be found in the cytosol and organelles, such as mitochondria and peroxisomes (Alexander et al., 2010). Based on the knowledge that ATM is kinase and affects the phosphorylation of PINK1 (Figure 1) which is indispensable for the biological function of Parkin activation (Gu et al., 2018). According to the latest research, ATM has been shown to regulate autophagy *via* tuberous sclerosis complex two or *via* the phosphorylation of hypoxia-inducible factor 1α. Furthermore, the role of ATM in mitophagy was confirmed in ATM-proficient cells and ATM-deficient cells with the spermidine-induced mitophagy model. Specifically, spermidine, which has a protective function against oxidative damage caused by hydrogen peroxide, induces mitophagy by causing mitochondrial depolarization (Eisenberg et al., 2009; Qi et al., 2016; Madeo et al., 2018). Depolarization of mitochondria means defective mitochondria, which causes accumulation of Pink1 and translocation of Parkin to damaged mitochondria leads to decreased mitochondrial mass in ATM-proficient cells (Qi et al., 2016). Whereas the loss of ATM results in mitochondrial abnormalities and the impairment of mitophagy, eliciting the accumulation of dysfunctional organelles (Valentin-Vega et al., 2012). Another research showed that by modulating DNA repair defects, it is possible to alleviate pathologies resulting from genome instability. PARP1 mediated immoderate poly ADP-ribosylation and it could alleviate the NAD^+^ levels which result in defective mitophagy (Fang et al., 2014, 2016). Recently, this mechanism has been demonstrated in neuroblastoma cells. ATM depletion results in a similar mitochondrial phenotype and mitophagy alteration. These phenomena could be partially rescued by NAD^+^ cofactor replenishment (Fang et al., 2016).

Ring finger protein 8 is a major factor in DNA DSB signaling pathway (Ma et al., 2011; Fang et al., 2016; Nakada, 2016). The RING finger-containing E3 ligase family is E3 ubiquitin ligases, and the specificity and ubiquitination type of substrate are determined by E3 ubiquitin ligase. In response to DNA damage, RNF8 mediates histone H2A and H2B mono-ubiquitination and facilitates the propagation of the DNA damage, the processes indispensable for DNA damage repair and activation of cell cycle checkpoint, and necessary to maintain genomic stability (Wu et al., 2009; Bohgaki et al., 2010). The primary function of RNF8 is to convert the DSB signal caused by exogenous or endogenous factors such as ionizing radiation (IR) or ROS, respectively (Yan and Jetten, 2008). A few recent reports confirmed the presence of RNF8 in the mitochondria; it was previously identified as a nuclear E3 ligase involved in non-homologous end-joining DNA damage repair. Ubiquitin receptors play the role of binding and transferring ubiquitinated cargo to the phagophore, initiate mitophagy (Husnjak and Dikic, 2012). In addition, it has been reported that 53BP1 (Figure 1), a DDR protein, is involved in damaged mitochondria clearance through mitophagy (Youn et al., 2017). Furthermore, it has already been reported that RNF8 is required for 53BP1 recruitment (Shao et al., 2009). These findings provide clear evidence that RNF8 can induce a DDR through mitophagy.

As a tumor suppressor, p53 has many different functions. A functional DDR prevents cells from uncontrolled proliferation; however, mutations could affect genes that control the DDR of the cell. DDR controls the biological signal a cell can enter and proceed through the cell cycle (Bartek and Lukas, 2007). p53 plays a crucial role in regulating cellular proliferation in response to DNA damage. Usually, p53 is a short-lived protein, and the activity of p53 is very strictly regulated through various processes including transcriptional and translational regulation and post-translational modifications (Honda et al., 1997). Moreover, p53 is rapidly ubiquitylated by mouse double minute 2 homolog (MDM2) and is subsequently targeted for degradation by the ubiquitin-dependent proteasomal system (Momand et al., 1992). However, when DNA damage occurs, p53 is stabilized by the DDR signaling system. In that particular case, p53 acts as a transcription factor and regulates the expression of target genes of p53 through a response factor in the promoter of that gene (Beckerman and Prives, 2010). The activity of p53 is also regulated by a number of post-translational modifications. As is widely known, p53 is involved in many signaling pathways and is known to have a bidirectional role in regulating autophagy. p53 plays a role in inducing the autophagy mechanism by activating AMPK, and also plays a role in inhibiting the autophagy mechanism by inhibiting the PI3K/Akt signaling pathway through the increase of PTEN expression (Zhang et al., 2015). Unlike ATM and RNF8, p53 binds to the RING0 region of Parkin, which interferes with the mitophagy process involved in Parkin, and affects mitochondria quality control, biosynthesis, kinetic regulation, and cellular redox homeostasis (Jung et al., 2017; Korolchuk et al., 2017). These results confirmed that mitophagy was increased by the downregulation of p53 in both *in vivo* and *in vitro* experiments using bone marrow mesenchymal stem cells (Zhang et al., 2020).

### ER-phagy

After translation, newly synthesized proteins enter the ER lumen and are structured according to their characteristics. If the protein modification process in the ER has trouble such as protein misfolding, a process called unfolded protein response (UPR) occurs that activates the intracellular signaling pathway for cells to maintain homeostasis. ER performs several functions, including folding of protein molecules and transporting proteins synthesized from vesicles to the Golgi apparatus. Unfolded protein, disturbance of redox or calcium regulation, and glucose deficiency trigger UPR and activate ER stress response (Kober et al., 2012). Recent clinical and preclinical studies suggest that ER stress is related to various metabolic diseases such as insulin resistance, diabetes, obesity, non-alcoholic fatty liver disease, and atherosclerosis. Therefore, proper regulation of ER stress can be a way to prevent the causes of various diseases. The ER stress pathway generated by UPR is closely linked to biological pathways such as cell survival, proliferation, autophagy, and apoptosis (Jäger et al., 2012; Ciechomska et al., 2013). While damaged mitochondria undergo targeted removal *via* mitophagy, as a result of ER stress, the portions of the ER are sequestered in autophagosomes in the process called ER-phagy, the type of selective autophagy to survive in severe ER stress condition. In general, ROS, one of the most representative factors known to cause DNA damage, induces ER stress. Several studies have reported that biological processes caused by ER stress are closely related to DNA damage (Groenendyk et al., 2010; Dicks et al., 2015). DNA damage induces the extension of tubular ER to facilitate ER-mitochondria signaling, thereby promoting apoptosis, a common mechanism of DDR (Zheng et al., 2018). Also, ER stress modulates the p53-related signaling pathway, leading to G2 arrest, thereby promoting apoptosis (Mlynarczyk and Fåhraeus, 2014). Studies are underway to reveal the link between DNA damage and ER stress, and it is known that p53 and C/EBP-homologous protein (CHOP) mainly regulate the life cycle of cells in ER stress conditions. p53 and CHOP are transcription factors and monitor genome integrity and stability. CHOP is upregulated by UPR, which is related to ER stress and generates ER stress response such as autophagy (Bernales et al., 2006; Hu et al., 2018).

Selective autophagy, ER-phagy, involves the formation of autophagosomes made from the ER membrane. ER is involved in the formation of autophagosome, and damaged ER is engulfed by itself by ER-phagy. Since ER-phagy also plays a role in removing the superfluous part from ER, ER-phagy activated against UPR can be said to be very important to establish homeostatic control (Bernales et al., 2007). ER-phagy could be similar to the regulation of peroxisome number, the balance between peroxisome biogenesis and pexophagy, and to the control of mitochondrial homeostasis (Dunn et al., 2005; Kundu and Thompson, 2005). TFEB and TFE3, nutrient responsive transcription factors, increase the expression of ER-phagy receptor reticulophagy regulator 1 (RETREG1)/FAM134B to induce ER-phagy (Cinque et al., 2020). C53, a cytosolic protein, is involved in the formation of autophagosome under ER stress condition (Stephani et al., 2020). A lot of effort is being put into identifying the ER-phagy mechanism. ER stress caused by the accumulation of unfolded proteins is the activation of UPR via three transmembrane proteins: inositol-requiring enzyme 1 (IRE1), protein kinase RNA-like ER kinase (PERK), and activating transcription factor 6 (ATF6) (Hetz, 2012). IRE1 and PERK are autophosphorylated, and ATF6 is exported to the Golgi apparatus and undergoes additional proteolytic cleavages. The downstream signaling pathways of these three proteins regulate the translation and transcription of proteins involved in relieving ER stress, and act as important factors in each step of the ER-phagy process, which removes damaged ER (Song et al., 2018).

**FIGURE 2:**
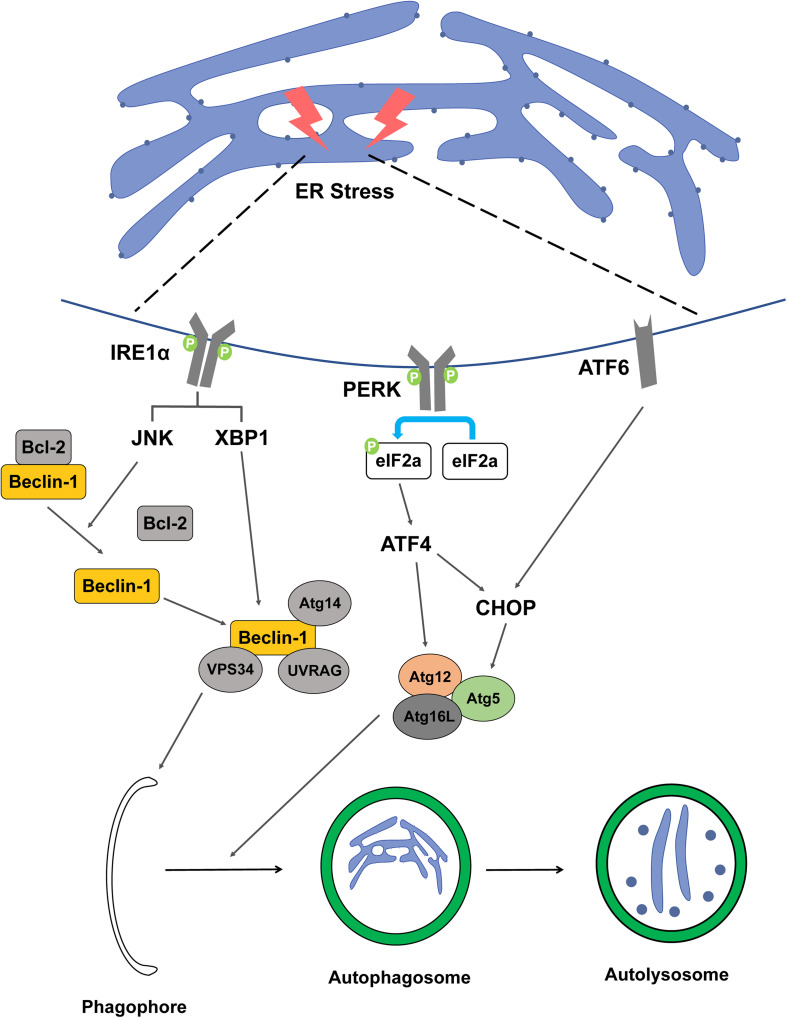
Schematic representation between ER stress factor and ER-phagy. The figure represents a signaling pathway of ER-phagy while damaged ER is engulfed by autophagosome. When ER stress is induced by DNA damage, the IRE1a, PERK, and ATF6 signaling pathways are activated, resulting in the formation of autophagosomes.

Inositol-requiring enzyme 1 is a sensor that activates UPR in the ER transmembrane and is a necessary factor to maintain ER and cellular function in animals and plants. IRE1 monitors ER homeostasis and determines whether the ER environment is appropriate or not by the luminal domain of the ER stress sensor present in IRE1. Thereafter, IRE1 triggers UPR through kinase and RNase cytoplasmic domains (Hetz and Glimcher, 2009; Hetz et al., 2011). In the UPR process, when a signal goes from the luminal to the cytosolic side of the ER, the signaling cascade proceeds and ER swelling occurs. The connection between ER-phagy and IRE1 is not well understood, however, there are experiments to elucidate the mechanism between the two. Epr1, a soluble ER-phagy receptor, was severely diminished in Δ*ire1* indicating that Epr1 upregulation requires Ire1 (Zhao et al., 2020). In another study, an experiment using 3-methyladenine or *Atg5* knockdown, which can inhibit autophagy, resulted in the finding that inhibition of autophagy can inhibit the IRE1 pathway of UPR (Liao et al., 2019). It can be said that the presented study results well explain the mechanism between IRE1 and ER-phagy. In mammalian cells, vesicle-associated membrane protein-associated proteins (VAPs) are involved in autophagosome formation by recruitment of the ULK1 complex and promote the connection between the ER and the autophagosome membrane (Bissa and Deretic, 2018). IRE1 forms a complex with tumor necrosis factor receptor-associated factor-2 (TRAF2) and apoptosis signal-regulating kinase-1 (ASK1), resulting in the downstream activation of stress kinase Jun-N-terminal kinase (JNK) that promotes autophagy (Ron and Hubbard, 2008; Gardner and Walter, 2011). Bcl-2 phosphorylated by JNK becomes an activated form and the Beclin-1/Bcl-2 complex is not formed. Free Beclin-1, which cannot form a complex with Bcl-2, forms a complex with vacuolar protein sorting 34 (VPS34) and is involved in the nucleation stage of autophagy (Pattingre et al., 2005). In addition, X-box binding protein-1 (XBP-1) is also activated by IRE1, promoting the transcription of Beclin-1, which is involved in autophagy induction (Figure 2; Hetz et al., 2009).

Protein kinase RNA-like ER kinase is one of the major transducers of ER stress and participates in the regulation of basic cellular functions. Autophagy and apoptosis signals induced by ER stress can be transmitted through the PERK signaling pathway, which has a switch mechanism to regulate cell death or cell survival (Liu et al., 2015). PERK activates activating transcription factor 4 (ATF4), a major transducer, by inhibiting the general protein translation process through phosphorylation of eIF2α (Rutkowski and Hegde, 2010; Hetz, 2012). ATF4 transcriptionally regulates Atg12, and ATF4-mediated CHOP activation transcriptionally induces Atg5, which includes the elongation process during autophagy (B’Chir et al., 2013; Kang et al., 2017). ATF4 upregulates light chain 3 (LC3), which plays a key role in the elongation and maturation of autophagosomes. LC3 interacts with the receptor of ER to selectively recognize defective ER and recruitment to proceed with ER-phagy (Figure 2). In addition, PERK, like IRE1, activates Beclin-1 to regulate the induction and nucleation stages of ER-phagy (Deegan et al., 2013).

Activating transcription factor 6 is a transcription factor and is transported to the Golgi apparatus when ER stress occurs, and then the N-terminal cytosolic domain is cleaved and moved to the nucleus. ATF6 moved to the nucleus binds to the ATF-cAMP response element and promotes transcription of ER stress response factors such as *XBP-1* and *CHOP* (Adachi et al., 2008; Guo et al., 2014). The activation of ER stress occurs *via* the eukaryotic translation initiation factor 2 alpha kinase 3 (EIF2AK3) and ATF6 UPR pathways, but not the ER to nucleus signaling 1 (ERN1)-XBP1 pathway, along with the upregulation of downstream signaling pathway both ATF4 and DNA damage inducible transcript 3 (DDIT3) (Wang et al., 2018). The ATF6 signaling pathway is required for the activation of ER-phagy (Figure 2). ER-phagy proceeds in the same system as general macro-autophagy and ATF6 is involved in this process under ER stress (Song et al., 2018). In addition, ATF6 induces the expression of death-associated protein kinase 1 (DAPK1), a kinase that phosphorylates Beclin-1 to form a phagophore, and interacts with C/EBP-β to not only participate in ER expansion but also contribute to the ER-phagy process (Gade et al., 2012).

### Ribophagy

Crosstalk between DDR and ribosome biogenesis has recently been reported. When the activated ATM n recruits NBS1 (Nijmegen Breakage Syndrome protein 1) to the nucleolus, this complex inhibits rDNA transcription (Larsen et al., 2014). This regulatory mechanism has a crucial role in the conservation of the stability of the rDNA genome which has a highly repetitive and actively transcribed feature. The ATM/NBS1/Treacle response is the particularly important response in the rDNA replication process. This response leads to forming DNA:RNA hybrids or R-loops and breakage of DNA double-strand (Pelletier et al., 2018). Thus, the ATM/NBS1/Treacle response is maintained at an appropriate level with the impaired ribosome synthesis checkpoint-p21 response to protect against DNA damage, which suggests that ribosome biogenesis in replicative stress is important (Pelletier et al., 2020). In addition, it has been found that ROS, one of the representatives DDR agents, not only attacks ribosome components but also affects ribosomes indirectly by altering the activities of ribosome-modifying enzymes. Ribosome modification by ubiquitin is one such example (Shcherbik and Pestov, 2019).

The molecular mechanisms of ribophagy have not been fully elucidated yet. Most studies have been performed using yeast models, and only a few studies have been conducted in mammalian models. Specific regulation of ribophagy is mediated by the deubiquitination of listerin E3 ubiquitin protein ligase 1 (Ltn1) by the ubiquitin carboxyl-terminal hydrolase 3 (Ubp3)/brefeldin A sensitivity 5 (Bre5) complex. A previous study showed that DNA damaging reagents induced the activity of the Ubp3/Bre5 complex, supporting the significance of ribophagy as a DDR (Bilsland et al., 2007). In addition, Ltn1 has been reported to recover the production of misfolded proteins derived from damaged mRNA (Yan et al., 2019), while nuclear fragile X mental retardation-interacting protein 1 (NUFIP1) was shown to serve as the major receptor of ribophagy machinery through specific binding to LC3B *via* the LIR motif within the NUFIP1. NUFIP1 targets the 60S ribosomal subunit; however, the actual ligand for this mechanism is not known and still needs further investigation (Wyant et al., 2018). To find out the mechanism of ribophagy, an experiment was conducted using Purkinje cells (PC), and macro-segregation of nuclear components and heterochromatinization were observed in these cells. This indicates a serious dysfunction of nuclear and extranuclear transcription, and it was found that free polyribosomes are replaced by monoribosomes (Baltanás et al., 2011). These monoribosomes were closely wrapped and appeared isolated into cytoplasmic compartments bound by sequestered rough endoplasmic reticulum (RER) cisterns and known as autophagosomes (Kraft et al., 2008). Accumulation of DNA damage in PCs of PC degeneration (pcd) mice results in nucleosome destruction, polyribosomal and monoribosomal autophagic degradation. This observation suggests that autophagy-related pathways are involved in the selective degradation of ribosomes and can be said to be a study to investigate the mechanism of ribophagy (Baltanás et al., 2011).

In the case of ribophagy, the role in yeast was first identified. Ubp3 in Yeast forms a complex with Bre5, a pivotal positive regulator (Li et al., 2005). The Ubp3/Bre5 complex is responsible for a wide variety of intracellular functions such as transcription elongation (Kvint et al., 2008), DNA repair by non-homologous end joining (Bilsland et al., 2007), and protein kinase C-mediated signaling (Wang et al., 2008). Another Ubp3/Bre5 complex plays a role in the autophagy process. It regulates the cytoplasm-to-vacuole targeting pathway through its action on Atg19, as well as the degradation of mature ribosomes that occur in starvation situations (Kraft et al., 2008). Another factor known to regulate ribophagy, Ltn1, is a protein known to perform ribosome-associated quality control (Bengtson and Joazeiro, 2010). In the absence of Ltn1 alone, it cannot directly affect the ribophagy pathway, but in the absence of Ubp3, it regulates the ribophagy pathway involving Ubp3/Bre5 complex instead (Ossareh-Nazari et al., 2010).

**FIGURE 3:**
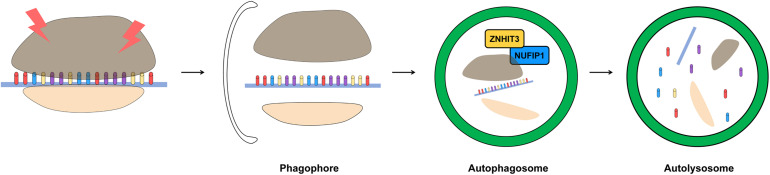
Schematic representation of removing damaged ribosome by ribophagy. The accumulation of damaged ribosome is removed by ribophagy. For the most part, the ribosome is related to RNA but ribosome biogenesis is affected by DNA. DNA damage interrupts ribosome biogenesis and decreases the stability of rDNA. ZNHIT3 and NUFIP1 have been suggested as a major factors of ribophagy induction in mammalian cells.

Ribophagy has recently been identified in mammalian cells. NUFIP1, an autophagy receptor for ribosomes, is required for ribophagy. NUFIP1 has an LIR motif and can bind to LC3 and forms a heterodimer with zinc finger HIT domain-containing protein 3 (ZNHIT3) to participate in the ribophagy process (Figure 3; Quinternet et al., 2016). It is known that NUFIP1-ZNHIT3 has a high probability of interacting with the 60S ribosomal subunit, and further studies are needed for detailed this pathway (Klinge et al., 2011). In the cytoplasm, NUFIP1 interacts with LC3 and transfers ribosomal cargo directly to the autophagosome. The degradation of ribosome induced in a starvation environment is accomplished through ribophagy, and this process depends on the capacity of NUFIP1 to bind to LC3 (Wyant et al., 2018). However, a ribosomal factor which interacted with NUFIP1 has not yet been identified, so further research is needed.

## Clinical Roles of the Autophagic Organelles in DDRs

### Molecular Pathology of DDR-Related Autophagic Organelles

Defects in DNA repair pathways induce the modification of DNA, and if this modification continues, mutations may accumulate and defects in polymerization and transcription of DNA or RNA may occur. These defects in the DNA repair mechanism induce apoptosis and aging, which could soon become a starting point for various diseases (Tiwari and Wilson, 2019). Many studies have shown that various diseases are caused by defects in the DNA repair system. Diseases caused by these defects are mostly related to aging and cancer. Furthermore, recent studies have shown that defects in the DNA repair system are known as a new cause of diseases associated with polyglutamine disease, such as Huntington’s disease, spinocerebellar ataxias, and other neurodegenerative disorders (Qi et al., 2007). Here, we summarize the latest research that connects diseases and autophagy in each subcellular organelle (mitochondria, ER, and ribosome), with the defects in the DNA repair system.

Mitochondrial dysfunction is associated with numerous biological phenomena including cancer. PINK1 is one of the representative factors related to mitochondrial function. Aging is known to be a major factor in causing cardiovascular disease (Liang and Gustafsson, 2020) and is also known as a major reason for age-related diseases such as Alzheimer’s disease (AD), Parkinson’s disease (PD), and Huntington’s disease (HD), and amyotrophic lateral sclerosis. Such aging proceeds according to biological conditions such as oxidative damage, telomere length, and mitochondrial dysfunction, and it is known that mitophagy is involved in regulating these abnormal environments (Tran and Reddy, 2020). When mitochondria are damaged, it is widely known to affect aging and neurodegeneration. As the name of Parkin, this protein is widely known as the protein associated with PD. Neurodegeneration in PD is related to mitochondrial dysfunction, and recently, studies have shown that PINK1/Parkin-dependent mitophagy responding to mitochondrial damage is associated with PD (Malpartida et al., 2021). Besides, there has been a study on the pathogenesis of PD through the association between α-synuclein aggregation and neuroinflammation (Liu et al., 2019). Mitochondrial dysfunction was also observed in a nucleotide excision DNA repair disorder with severe neurodegeneration (Fang et al., 2014). Synthetically, mitophagy could be a promising strategy in the treatment of neurodegenerative disorders. PINK1 has been reported as a gene whose expression is increased by overexpression of PTEN, a representative tumor suppressor in cancer cells. PINK1 has also been shown to be downregulated in the absence of PTEN (Unoki and Nakamura, 2001). Research over the past decade has shown that PINK1 is implicated in various cellular functions such as cell survival, stress resistance, and mitochondrial homeostasis in cancer cells (O’Flanagan and O’Neill, 2014). PTEN is one of the most frequently mutated genes in several cancers, including glioblastoma, endometrial, breast and prostate cancers (Steck et al., 1997). Since it was found that PINK1 is regulated by PTEN, studies on the relationship between PINK1 and PI3-kinase/Akt signaling system have been intensively conducted. PTEN-induced increase in PINK1 and decrease in PI3K/AKT have been found in PD and several cancers, suggesting that PINK1 plays an important pathological function (MacKeigan et al., 2005). PINK1 has an important pathophysiological function, so it could be a promising drug target in diseases such as neurodegeneration, aging, and cancer.

*Ataxia-telangiectasia mutated* gene mutations cause the development of Ataxia-Telangiectasia (AT). AT is a rare genetic disorder that affects body systems, including the nervous and immune systems. All AT patients have mutations in the *ATM* gene. Dysfunction in DNA damage repair, apoptosis, and cell cycle is due to mutations in the *ATM* gene. BNIP3, acting as an inducer of mitophagy, showed low expression levels in AT cells, suggesting that the mitophagy pathway malfunctions when the *ATM* gene is mutated (Sunderland et al., 2020). It is known that AT patients also have an increased risk of cancer due to the loss of ATM function (Friedenson, 2007). Usually, a heterozygous mutation of ATM is found in cancer. According to the Somatic Mutation Catalog of Cancer (COSMIC), the frequency of *ATM* gene mutation is 0.7% in 713 ovarian cancers, 0.9% in central nervous system cancers, 1.9% in 1,120 breast cancers, 2.1% in 847 kidney cancers, 18% in 74 colon cancer, 7.2% in 1,040 lung cancers, and 11.1% in 1,790 hematopoietic and lymphoid tissue cancers. In pancreatic cancer, one of the representative malignant carcinomas, it has been reported that 6.4% of 5,234 patients had an ATM mutation in germ cells or somatic cells (Cremona and Behrens, 2014).

RNF8 is an enzyme that plays an important role in DNA repair and an appropriate level must be maintained. It has been reported that RNF8 causes genomic instability, tumorigenesis, and malignant tumors when RNF8 exceeds appropriate levels and accumulates excessively (Singh et al., 2019). Additionally, RNF8 is an associated partner of estrogen receptor α (ERα) and activates ERα-mediated responses in breast cancer cells *in vitro*. As with the results *in vitro*, it was confirmed that RNF8 was positively correlated with ERα in breast cancer patient tissues (Wang et al., 2017). Conversely, even when the expression of RNF8 is reduced, pathological issues arise. It has been reported that the expression of RNF8 decreases when infection or disease progresses, causing genomic instability in adult T-cell leukemia (Zhi et al., 2020). Recent studies also reported that decreased expression of RNF8 increases genomic instability and vulnerability to tumorigenesis in prostate cancer (Li et al., 2010). These results indicate that RNF8, a key factor of DNA repair, has potential as a novel tumor suppressor.

p53 actually has many functions, and disease can occur if the level of this protein is not properly regulated. Recent studies have shown that mitophagy upregulates hepatic cancer stem cells (CSCs) by inhibiting p53, well known as a tumor suppressor. Transcription factors are important for maintaining the stemness and self-renewal capacity of CSCs. For example, when phosphorylated p53 binds to the NANOG promoter, it is important to reduce the hepatic CSC population by preventing transcription factors OCT4 and SOX2 from upregulating the expression of NANOG. Mitophagy regulates the ability of p53 to maintain hepatic CSCs, providing an explanation why autophagy is necessary for promoting hepatocarcinogenesis. p53 downregulates NANOG and is eliminated together with mitochondria by mitophagy (Liu et al., 2017).

Cellular stress can disturb the protein-folding functions of ER, driving many types of cancer cells to activate the UPR as the means of sustaining malignant growth while retaining viability (Wang and Kaufman, 2014). In estrogen receptor α positive (ERα^+^) breast cancer, the UPR in general, and XBP1 in particular, contribute to acquired resistance against anti-endocrine therapy (Clarke and Cook, 2015). A recent study revealed that triple-negative breast cancer (TNBC) cells highly depend on IRE1 to adapt their ER to *in vivo* stress and to accommodate the tumor microenvironment to promote malignant growth. Other studies have also linked the IRE1α–XBP1s pathway to the MYC, transcription factor and a potent driver of proliferation in TNBC, prostate cancer, and B-cell lymphoma. In addition, IRE1 mutations in glioblastoma multiforme were recently reported, with one mutated form of IRE1 characterized by elevated regulated IRE1-dependent decay activity and reduced ability to form tumors *in vivo* (Sheng et al., 2019).

Recent studies investigating the role of PERK have suggested both pro- and anti-tumorigenic functions. PERK signaling pathway is used when both tumor initiation and expansion to maintain redox balance for facilitating tumorigenesis (Bobrovnikova-Marjon et al., 2010). The activation of PERK-eIF2α axis causes reduction of proliferation and increase of apoptosis and this pathway during the loss of intestinal epithelial stemness and enforced differentiation (Heijmans et al., 2013). Subsequent studies demonstrated that PERK signaling mediates arrest in the G1 phase. Activation of PERK and phosphorylation of eIF2α suppress protein translation, including cyclin D1 (Hamanaka et al., 2005). Because of its short half-life, expression of cyclin D1 is greatly decreased during ER stress. Decreased cyclin D1 expression results in the impaired activity of cyclin D1-CDK4 complex, thereby ensuring cell cycle arrest at the G1 phase (Brewer et al., 1999).

There is a significant correlation between the expression of ATF6 and inhibitor of DNA binding 1 (ID1) in high-grade serous ovarian cancer tissues. Furthermore, patients with high expression of ATF6 or ID1 have a resistance to platinum treatment and the overall survival rate was low (Meng et al., 2020). In addition, ATF6 was found to be highly expressed in areas undergoing pre-cancerous atypical change in both non-ulcerative colitis (UC) and UC-associated CRC, and this can be used to determine the grade level of UC patients (Hanaoka et al., 2018). In addition, in the mouse model with high ATF6 expression, after 4 days of the activation of ATF6, the bacteria were close to the colon epithelium, the cell proliferation rate was faster, and 100% tumors developed within 26 weeks. It suggests that these alterations are early events activation of ATF6 downstream. These findings suggest that activated ATF6 induces an innate immune response to promote colorectal tumorigenesis (Lavoie and Garrett, 2018).

### Pharmacological Approach to DDR-Related Autophagic Organelles

Based on the pathological mechanisms induced by defects in the DNA repair system related to autophagy for each organelle described above, we summarize here the research treatment strategies for each disease, as well as the potential for each factor as a drug target (Table 1).

**TABLE 1:** Pharmacological targeting of DDR factors.

**Target**	**Compound**	**Stage**	**References**
ATM	KU55933	Preclinical	Hickson et al., 2004
	KU60019	Preclinical	Golding et al., 2009
	CP466722	Preclinical	Rainey et al., 2008
	Caffeine	Preclinical	Blasina et al., 1999
	Wortmannin	Preclinical	Sarkaria et al., 1998
	TPA	Preclinical	Truman et al., 2005
	Vitamin B3	Phase 2	NCT03962114
RNF8	Corilagin	Preclinical	Qiu et al., 2019
p53	STIMA-1	Preclinical	Zache et al., 2008
	APR-246	Preclinical	Bykov et al., 2002; Bykov et al., 2005b
	CP-31398	Preclinical	Foster et al., 1999
	MIRA-1	Preclinical	Bykov et al., 2005a
	RITA	Preclinical	Issaeva et al., 2004; Jones et al., 2012
IRE1	B-109	Preclinical	Tang et al., 2014
	STF-083010	Preclinical	Chen et al., 2018
	KIRA6–8	Preclinical	Ghosh et al., 2014; Morita et al., 2017
	Toyocamycin	Preclinical	Ri et al., 2012
	Doxorubicin	Preclinical	Jiang et al., 2016
PERK	GSK2606414	Preclinical	Mercado et al., 2018
	GSK2656157	Preclinical	Atkins et al., 2013
ATF6	Ceapin-A7	Preclinical	Gallagher and Walter, 2016
	PBA	Early Phase 1	NCT04041232

In the latest research, several studies showed increased cellular sensitivity toward genotoxic agents by modulating the ATM signaling *via* the specific ATM kinase inhibition. This drug induced tumor senescence in breast, lung, and colon carcinoma cell lines and verified the ATM/ATR signaling pathway to be constitutively active in cancer cells. Furthermore, the addition of the ATM kinase-specific inhibitor KU55933 (Hickson et al., 2004) or another ATM/ATR dual inhibitor CGK733 (Alao and Sunnerhagen, 2009), caused the increase of apoptosis in these cancer cells. Interestingly, although this treatment was cytotoxic to these cells, it did not lead to apoptosis in the normal senescent human fibroblasts. ATM expression inhibition, through ATM gene silencing using shRNA or siRNA, is another strategy (Guha et al., 2000). Furthermore, using the KU55933 treatment, blocking the function of ATM in these cancer cells caused the increase of radiosensitivity because the cells cannot repair the damage caused by homologous recombination repair (Neijenhuis et al., 2010). 12-O-tetradecanoylphorbol 12-acetate (TPA), the protein kinase C activator, functions to decrease the cellular level of the ATM. TPA and ATM are related to each other in the radiosensitization of an otherwise radioresistant human prostate cancer cell line and in the induction of apoptosis. Treatment of cells with TPA decreases ATM activity and increases the level of apoptosis that induces the regulatory enzyme ceramide synthase, resulting in induction of apoptosis after IR treatment (Truman et al., 2005). Another advantage of targeting ATM such as KU-60019 is revealed in HD. ATM inhibitors have not been used before to treat brain disease conditions *in vivo*. But this research presents the reasons for future pharmacokinetic studies (Lu et al., 2014).

Ring finger protein 8 is known as a promising target for chemotherapy because it is aberrantly expressed in many breast cancer patients, promotes tumor metastasis, and plays a key role in the DDR pathway. Targeting of RNF8 not only suppresses or eliminates the metastatic capacity of cancer cells but also increases the sensitivity of cancer cells to anticancer drugs upon depletion of RNF8, which can greatly improve the efficacy of anticancer drugs (Kuang et al., 2016). For example, corilagin targeting RNF8 effectively inhibits cell proliferation of esophageal squamous cell carcinoma (ESCC) and induces apoptosis. This compound caused significant DNA damage in ESCC cells and significantly attenuated the RNF8 expression through the ubiquitin-proteasome pathway, blocking the DNA damage repair pathway and causing cell apoptosis (Qiu et al., 2019).

SH-group targeting and induction of massive apoptosis (STIMA-1), a low molecular weight compound with some structural similarity to CP-31398, known as a p53 inhibitor, stimulates the binding of mutant p53 to DNA *in vitro*, induces the expression of the p53 target protein, and can cause cell death in tumor cells expressing the mutant p53 (Zache et al., 2008). A small molecule called p53 reactivation and induction of massive apoptosis-1 (PRIMA-1) was developed to restore the original function of the tumor causing mutant p53. The effect of PRIMA-1 and its derivative, PRIMA-1^MET^ (APR-246 was verified *in vitro*, and it was identified as a compound that specifically inhibits the growth of p53 mutant tumor cells (Bykov et al., 2002). Similarly to PRIMA-1 and mutant p53 reactivation and induction of rapid apoptosis (MIRA-3), which also rescued the function of mutant p53 *via* thiol modification in the DNA binding domain, PRIMA-1 upregulated p53 activity by recovering sequence-specific DNA binding and facilitated the mitochondrial dependent intrinsic apoptosis program through the activation of caspase-2 (Shen et al., 2008). Upon binding to p53, RITA (also known as NSC 652287) also reactivates it and promotes cell death by interfering with its interaction with MDM2. The IC_50_ value of RITA depends on which cancer cell it is, but growth inhibition is clearly more effective (Issaeva et al., 2004).

Endoplasmic reticulum stress can be induced by DDR, and autophagy can be activated by factors involved in ER stress, IRE1, PERK, and ATF6. Activated autophagy can be involved in a number of disease factors, and controlling ER stress factors can be a method of treatment. Drugs that target IRE1, an ER stress factor, to give clinical effects have been suggested *in vitro* and *in vivo*. Representatively, Studies have shown that RNA attenuator, KIRA, can inhibit IRE1. KIRA 6, one of the KIRAs, was found to inhibit IRE1 and promote cell survival (Ghosh et al., 2014). Another KIRA, KIRA 7, has been reported to decrease UPR signaling and protect lung fibrosis by inhibiting IRE1. Finally, it was found that KIRA8 is a compound having a structure different from KIRA7 but can inhibit pulmonary fibrosis by inhibiting IRE1 (Thamsen et al., 2019). These suggestions imply that proposed drugs can inhibit autophagy and some diseases, which is increased by IRE1.

The control of other ER stress factors, PERK and ATF6, has also proven the effectiveness of treatment in several papers. GSK2656157, an ATP-competitive inhibitor that lowers the enzyme activity of PERK, is used as an inhibitor of PERK and has been found to affect tumor growth. By administering this drug orally, it can target PERK and effectively control tumors under microenvironment stresses such as hypoxia or nutrient starvation (Atkins et al., 2013). GSK2606414, a drug targeting PERK in the same way as GSK2656157, shows that oral administration can effectively prevent PD, a disease that causes clinical symptoms by killing dopaminergic neurons in the midbrain of the brain, under ER stress. However, in the case of GSK2606414, it has been reported that pancreatic toxicity occurs, so continuous experiment is needed (Mercado et al., 2018). Studies have shown that drugs targeting ATF6, another ER stress factor, are effective in the treatment of diseases. As an example, Ceapin, a type of pyrazole amides, inhibits ATF6 signaling, which leads to the alleviation of ER stress (Gallagher and Walter, 2016).

## Conclusion

Until now, the connection between DDR and autophagic organelles has been evaluated; however, there is a limited number of studies investigating DDR factors related to selective autophagy (mitophagy, ER-phagy, and ribophagy). In this review, we summarize not only the role of autophagy in DDR, but also the pathophysiology of a wide range of diseases, as well as potential pharmacological regulators affecting both DDR factors and selective autophagy. Since autophagy emerges as a promising drug target, our understanding of its exact mechanisms in DDR is crucial when targeting pathologies, such as cancer, liver diseases, and brain disorders. Therefore, the inactivation of DDR factors by small molecule inhibitors should provide a new strategy for the treatment of diverse diseases. Thus, further research of the molecular mechanisms related to the regulation of DDR and autophagy is one of the ways to increase treatment efficiency. We hope that this review presented a far deeper understanding of DDR and autophagic degradation of organelles and could guide researchers pursuing clinical investigations in this scientific field.

## Author Contributions

JK and BY: conceptualization and writing original draft preparation. JK, SL, HK, HL, KMS, HY, and BY: writing review and editing. BY: supervision and project administration. All authors contributed to the article and approved the submitted version.

## Conflict of Interest

The authors declare that the research was conducted in the absence of any commercial or financial relationships that could be construed as a potential conflict of interest.
